# Plant Phenotyping using Probabilistic Topic Models: Uncovering the Hyperspectral Language of Plants

**DOI:** 10.1038/srep22482

**Published:** 2016-03-09

**Authors:** Mirwaes Wahabzada, Anne-Katrin Mahlein, Christian Bauckhage, Ulrike Steiner, Erich-Christian Oerke, Kristian Kersting

**Affiliations:** 1 INRES-Phytomedicine, University of Bonn, Bonn, Germany; 2 Fraunhofer IAIS, Sankt Augustin, Germany; 3 B-IT, University of Bonn, Bonn, Germany; 4 CS Department, Technical University of Dortmund, Dortmund, Germany

## Abstract

Modern phenotyping and plant disease detection methods, based on optical sensors and information technology, provide promising approaches to plant research and precision farming. In particular, hyperspectral imaging have been found to reveal physiological and structural characteristics in plants and to allow for tracking physiological dynamics due to environmental effects. In this work, we present an approach to plant phenotyping that integrates non-invasive sensors, computer vision, as well as data mining techniques and allows for monitoring how plants respond to stress. To uncover latent hyperspectral characteristics of diseased plants reliably and in an easy-to-understand way, we “wordify” the hyperspectral images, i.e., we turn the images into a corpus of text documents. Then, we apply probabilistic topic models, a well-established natural language processing technique that identifies content and topics of documents. Based on recent regularized topic models, we demonstrate that one can track automatically the development of three foliar diseases of barley. We also present a visualization of the topics that provides plant scientists an intuitive tool for hyperspectral imaging. In short, our analysis and visualization of characteristic topics found during symptom development and disease progress reveal the hyperspectral language of plant diseases.

The plant phenotype is of importance to evaluate the performance of a crop as the interaction between a plant genotype and its environment^1^. Recently, phenotyping is defined as a set of methodologies and protocols to assess plant parameters at different scales^2^,^3^. Within this context, non-invasive sensors and computer based technologies demonstrated their potential to equip todays agriculture with tools to solve current and future challenges^4^. Especially the detection of plant diseases is an important task in crop production to avoid yield losses, and in plant breeding for the selection of diseases resistant genotypes. Today’s approaches to disease detection and planning of plant protection measures still very much rely on human experts and/or on prognosis models. Unfortunately, these scale badly to the growing amounts of data in plant phenotyping and are prone to human conformation bias. Barley, for example, may be affected by various foliar pathogens during the vegetation period, and significant quantitative and qualitative yield losses are caused by diseases like powdery mildew, net blotch and brown rust^5^. Each of these diseases causes characteristic symptoms and the need to improve and to automatize their monitoring in fields and/or greenhouses has led to an increasing adoption of technologies such as hyperspectral imaging. This kind of *sensor-based phenotyping* has already been proven successfully for monitoring physiological traits and plant genotype-specific responses to biotic and abiotic stresses^6^,^7^,^8^. Especially hyperspectral imaging data of individual plants or crop stands contains an enormous amount of information on their physiological and biochemical status^7^,^9^,^10^. The reflectance values of continuous wavebands of the electromagnetic spectrum are influenced by various plant characteristics; any kind of stress causes complex changes in the plants’ physiology and composition which, in turn, alters the spectral reflectance pattern (=spectral signature) of plants in the visible range (VIS, 400–700 nm), near-infrared (NIR, 700–1000 nm) and short wave-infrared (SWIR, 1000–2500 nm).

The work presented here is motivated by the insight that hyperspectral measurements can reveal relationship between the spectral reflectance properties of plants, and their structural characteristics and pigment concentrations, which are considerably influenced by biotic plant stress^3^,^11^. This indicates that phenotyping processes can benefit from hyperspectral data analysis and machine learning techniques which can uncover the characteristics of how plants respond to environmental stress. However, since stress reactions result from a complex web of interactions between the genotype and the environment^12^, common data analysis methods for plant phenotyping such as spectral vegetation indices run the risk of leading to an over-simplified or even misleading interpretation of spectral responses to stress as they consider only few distinct wavelengths. On the other hand, many advanced machine learning techniques extract “factors” or “features” from the data that are not “things” with a “physical” reality^13^. In turn they are often not easy-to-interpret for non-experts in machine learning. Consequently, next generation plant phenotyping and plant disease detection systems require comprehensive and reliable data analysis methods.

In this work, we propose an automated data mining approach that was adopted from the areas of natural language processing and text mining. There, probabilistic topic models have been proven to successfully capture content and underlying hidden topics of document collections and thus to help to organize, search, and understand large amounts of data^14^. Probabilistic topic model are known to allow for learning meaningful and interpretable representations of massive document collections. As illustrated in Fig. 1(A), given a corpus of text documents, topic models characterize each document using a small number of topics-the clusters. Topics are distributions over words estimated automatically from the documents, where semantically related words have higher probabilities (weights) within a topic. Due to the simple representation as distributions over words, topics are easy to interpret for human analysts. Consider e.g. a subset of Wikipedia articles. Figure 1(B) shows the topics discovered by latent Dirichlet allocation (LDA)^15^—arguably the most popular probabilistic topic model—represented as word clouds containing the most probable words per topic. As one can see, probabilistic topics indeed result in a meaningful short description; in our case, they are easily interpretable as “Music”, “Color”, or “Education”.

Other common approaches for decomposing large data matrices into latent components include principal component analysis (PCA), non-negative matrix factorization (NMF), and archetypal analysis (AA), among others. PCA determines a factorization that retains as much variation in the data as possible^16^ and is often used for data compression as it reduces noisy and redundant information. NMF^17^ considers matrices with non-negative entries and results in part-based representation of the data. AA^18^,^19^ explains the data in terms of combinations of extreme observations, which are more distinct and hence are more interpretable by human analysts. All these methods implicitly consider a document as a single point in an abstract high dimensional data space. Topic models, in contrast, can provide interpretable representations, which have statistical properties that correspond to those of semantic networks, produced by humans^20^. Furthermore, although there is a connection between NMF and probabilistic topic modeling^21^, NMF typically learns more incoherent topics than LDA^22^. Moreover, the LDA model is easier to explain as it is a generative model: word distributions compromises topics, and a document is drawn from a specific mixture of topics. In turn, the latent components determined by LDA are closer to the probabilistic “*topic metaphor*” and do not require to reify the “*basis vectors*” found by NMF. This is essential as the application domain we consider in this work is interdisciplinary and requires scientist with different background to work together. Compared to methods that represent data in terms of extremes or archetypes, LDA can be considered as part-based archetypal analysis. Thus, the topics are extreme distributions in a space spanned by the words in the vocabulary. This view corresponds to the geometric interpretation of LDA as an analysis of data points distributed on a latent simplex^15^ and, in turn, allows for representing the data as points in the simplex spanned by the topics.

Our analysis is based on hyperspectral images of plants in the visible and near infrared ranges. While there are often labels per images such as different genotypes or treatmens of plants, the hyperspectral signatures from single pixels on the images-the focus of our study-are typically not annotated. Hence, it is difficult-if not impossible-to directly employ classification approaches to gain insights into the important hyperspectral characteristics of plant disease progressions^4^,^23^. More importantly, the benefit of hyperspectral imaging for plant phenotyping goes beyond the classification of plant stress. Recent studies^4^,^24^,^25^ presented automated analysis pipelines for tracing effects of abiotic and biotic stress to crop plants. Within this context, probabilistic topic models are an intuitive and effective approach for automated, time and cost saving data analysis in order to obtain a deeper understanding of plant pathogen interactions. As we will demonstrate, this exploratory data analysis approach can provide new insights into processes during stress emergence and offers the ability to study how plant physiology is influenced during pathogen infection.

In order to extract meaningful topics, i.e. hyperspectral characteristics in terms of important wavelength × reflectance pairs, we propose to “documentify” the hyperspectral images, i.e., we first create “*documents*” out of hyperspectral signatures. To this end, we “wordify” waveband-energy values as illustrated in Fig. 1(C). Then, we discover hyperspectral characteristics by means of an efficient online approach to regularized LDA. Together, these steps allow one to automatically learn easy-to-interpret representations from large collections of hyperspectral images consisting of millions of pixels/signatures similar to representations used previously to analyze plants suffering from drought^26^. In other words, this approach provides spectral characteristics of plants affected by various foliar pathogens during the vegetation period. The corresponding topics describe the development of different plant diseases during pathogenesis, allow for an intuitive visualization of information from hyperspectral images, and provide new insights into the dynamics of plant diseases.

## Results

In this section, we present and discuss our experimental evaluation on barley plants during development of the foliar diseases powdery mildew, net blotch, and brown rust. The data set considered in this study consists of single barley leaves, recorded every other day after inoculation (dai) with hyperspectral imaging line scanner in the visible and near infrared (400–1000 nm) range^4^. Each hyperspectral image was represented as dense Λ × *N* matrix, where *N* denotes the number of pixels and Λ the number of spectral bands. Stacking all data matrices recorded during pathogenesis into a single matrix resulted in a data matrix with approximately 10 million columns or about 2 billion matrix entries (encoding the reflected energy at different spectral bands). Before determining the topics, we first created sparse matrices out of dense signature × pixel matrices using a wordification approach (see the Methods section). We then ran online regularized LDA for three datasets (for each disease) separately to obtain a set of highly relevant topics related to diseased as well as healthy barley plants. We stopped the online inference when each signature (document) was seen once for each data. Experiments were run on a standard Intel SixCore machine with 3.2 GHz and 16 GB main memory. It took approximately 1.5 hours to determine the topics for each disease dataset where the number of topics was set to *K *= 25. The Python implementation of online regularized LDA is freely available at https://github.com/mirwaes/sclda.

### Topic Labeling

After the models were learned and specific topics for each disease and healthy barley plants were obtained, we manually annotated the corresponding topics based on information from the literature and their relation to the plant health status. We identified eight classes for net blotch and powdery mildew and six for brown rust. These are visualized in Fig. 2 and summarized in Table 1. Since diseased plants show signs of stress only locally, they also contain examples of topics characteristic for healthy leaves, which can be found in classes 1 and 2 (green boxes) for all diseased plants. Furthermore, regularized LDA can also uncover the specific spectral characteristics at different stages of pathogenesis, as covered by the classes 3–7 for powdery mildew and net blotch, as well as in classes 3–6 for brown rust.

In contrast to previous works that considered full signatures to explain the disease progression (cf. Wahabzada *et al*.^4^ and references thereine), the topics considered here provide a part based representation covering important wavelength × reflectance pairs. For instance, class 1 has top words (specific wavelength × reflectance) in the range of 550 nm which is highly correlated to the chlorophyll content^10^,^27^, the most important pigments in living plants as they are necessary for photosynthesis. The topics which were labeled as diseased in the VIS range (e.g. red and brown boxes in Fig. 2) have top words between 550–700 nm, indicating the disease symptoms such as small necrotic tissue for net blotch, chlorotic spots in early rust development and auburn pustules, and fluffy mycelium and conidia distributed on the upper and lower leaf side for powdery mildew. This caused an overall increase of reflectances which was also observed by^28^,^29^ or by^30^ for *Cercospora beticola* in sugar beet.

### Disease Dynamics

The evaluated topics exhibit a specific location on diseased leaves. Therefore, each topic could be connected to a specific symptom and the sum of all topics explains the spectral variability within barley leaves. Moreover, localization and probability of topics over time are highly dynamic. This is visualized in Fig. 3(A) for the example of barley leaves infected with powdery mildew 6, 10 and 14 dai (days after inoculation). The first two topics represent the border of a powdery mildew pustule and topic three represents the center of powdery mildew colonies. With further pathogenesis the dominance and probability of the topics change to symptom development. Similar dynamics could be visualized for barley leaves with net blotch and brown rust. This accords with Mahlein *et al*.^30^ and Wahabzada *et al*.^4^ who previously described hyperspectral dynamics of diseased plants.

To compute the disease dynamics quantitatively, we determined the disease progression using the representations learned by regularized LDA. We automatically labeled each pixel *i* on the image as “diseased”, when the sum of probabilities of labeled diseased topics was greater than a threshold value of *ε* = 0.25. Then, for each diseased leaf we computed the relative number of diseased/necrotic pixel for 6, 10 and 14 dai. As shown in Fig. 3(B), there is a difference in the amount of affected pixels (=size of infected area on a leaf) for plants inoculated with different diseases. The number of pixels with disease topics is higher for leaves with powdery mildew than for those with other diseases (14 dai). Net blotch and brown rust show similar levels of infestation in the early stages. Brown rust caused tiny chlorotic spots appearing on the tissue, necrosis and loss of water occur only at later stages. Therefore, it has a lower level of colonization than net blotch and powdery mildew in the early stages.

### Relative Relevance over Time

In a next step we computed topic relevance at different stages of disease progression. To assess the age of disease symptoms, it is important to determine how likely it is to observe a particular topic at specific point in time during pathogenesis. Hence, we computed the relevance for a topic *k* using 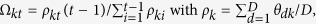 where *t* denotes the day after inoculation and *θ*_*d*_ is the topics representation of a document *d*. Note that we do not measure the appearance of the topics per pixel, as it was done in the previous section, but the relative increase in probability for each topic compared to the previous days. The word clouds in Fig. 2 show the results with respect to the increase in topic relevance 6, 10 and 14 dai. Here, the size of the text is proportional to the computed values Ω_*kt*_. The diseased/necrotic topics become more prominent at later stages, whereas the significance of healthy (green) topics is low.

The diseased/necrotic topics for powdery mildew in Fig. 2(A) have higher importance starting at day 6 after inoculation. This can be explained by the high amount of white mycelial colonies on the the relatively intact tissue that caused a high number of conidia produced^4^. Net blotch, on the other hand, showed early chlorosis and necrosis, causing structural and biochemical changes and necrotic tissue damage, as covered by the relevances in Fig. 2(B). Brown rust showed comparatively minor impact on barley tissue in early stages, which can be also deduced from Fig. 2(C). First chloroses appeared around 7 dai causing an increase of the relevance of topics covering *structural changes* and *pustule border*. However, rust spores started to rupture the epidermis 10 dai, causing an increase in importance of topics related to *sporulation*.

## Discussion

We present an automated, data-driven pipeline for extracting characteristic spectral regions of plants, infected by foliar pathogens. Effective analysis and interpretation of hyperspectral imaging data are still limiting factors for an implementation of sensor technologies into plant phenotyping or precision agriculture^31^,^32^. Probabilistic topic models, originating from text mining, were successfully adopted to analyze hyperspectral images of plants. Based on the proposed pipeline, it is possible to uncover the hyperspectral language of plant diseases, to visualize characteristic topics during symptom development, and to monitor disease progress. Detecting and utilizing information of the electromagnetic spectrum of plants, infected with pathogens, one can observe disease development during pathogenesis^33^. The proposed pipeline strikes a new path for plant phenotyping and characterization of early processes during pathogenesis by optical sensors. The word clouds, shown in Fig. 2, are an example of an interpretable summary of high dimensional data, elucidating processes and key aspects of pathogenesis. Compared to common data analysis approaches, multiple benefits are present. In contrast to vegetation indices, which are a correlated to biophysical plant parameters and are not disease specific^27^,^34^, the entire spectral information is utilized effectively. Wavelet analysis, which outperformed a range of spectral vegetation indices in a predictive model for chlorophyll content^35^, aims to provide meaningful quantitative information, but would hardly be capable to gather the entire complexity of up- and down regulated parameters during plant disease development. Classification methods such as Support Vector Machines or Artificial Neural Networks aim at differentiating among classes such as healthy or diseased plant tissue^23^,^32^. Here the results highly depend on the choice of features from hyperspectral images and could be a complementary methodology to our proposed probabilistic topic models, avoiding time intensive and error prone human labelling.

The hyperspectral language of plants assessed with the wordification approach corresponds to the phenotype of diseased plants and enables a highly accurate description of disease progression over time and in space. They result in hyperspectral topics that conform to plant physiological knowledge, allowing to characterize plant pathogen interactions. This is demonstrated in Fig. 2 and Table 1, showing the relevant hyperspectral topics and corresponding biochemical labels. A comparison of latent components found by different data mining methods from a hyperspectral image of a diseased plant is shown in Supplementary Fig. S1(A). Principal components obtained by PCA, for instance, can reveal the variations existing in the data, but the corresponding intensities are abstract values that make statistically sense, but they do not have a physical meaning. Other methods, such as NMF or simplex volume maximization^19^ (SiVM, a fast method for archetypal analysis), find components that are corrupted by noise or can not represent important parts of hyperspectral signature. The wordification approach, on the other hand, extracts latent part-based components that contain the reflectance intensities at different wavelengths, as shown in Fig. 2 and summarized in Table 1. They can be interpreted easily by domain experts. A comparison to a standard approach for topic models without regularization has revealed that the topics found by non-regularized LDA, shown in Supplementary Fig. 1(B), are not coherent. Furthermore, they are dominated by topics with lower reflectance intensities, which represent the healthy part of the leaf, while ignoring the variations of diseased spectra. This is another justification for the proposed fast regularized LDA, as it considers the short-range dependencies of hyperspectral words and produces coherent topics that can be associated with different leaf disease stages.

The hyperspectral topics and the resulting word clouds visualizes the underlying biophysical and biochemical processes during disease development. The identified topics belong to specific regions of disease different symptoms or/and to specific developmental phases. This aspect is in accordance to^30^,^36^, who found characteristic spectral signatures for symptoms of Cercospora leaf spot of sugar beet, in time and space. The prominence of a specific trait or developmental phase can be visualized intuitively by the presented wordification approach. Powdery mildew diseased tissue is covered by white mycelium colonies producing an increasing amount of conidia. Besides a development of characteristic powdery pustules, accompanying chlorosis can be read from the topic models. Net blotch and rust share the occurrence of chlorosis in early stages of disease development. Besides, net blotch causes early necrosis. Due to this specific necrotrophic aspect, topics, correlated to pigment degradation, water loss and cellular tissue damage are characteristic for net blotch infestation. As a biotrophic fungi, *P. hordei*, the causal agent of rust disease has a moderate influence into the host plant biophysiology. The relation among healthy and diseased tissue is well-balanced over time, sporulation specific topics appear at later time points. These observation are in accordance to hyperspectral dynamics of barley diseases visualized as single sketches and metro maps of plant diseases by^4^.

Our work provides several interesting avenues for future work. Next to experiments under field conditions, one should aim at even further improving the topic quality, for instance, by applying hierarchical, (semi-) supervised and relational versions of topic modeling. The models may be used to identify the most relevant time when biologists have to gather samples for invasive, molecular examinations. Active LDA approaches could be employed to speed up computations even further. This would also allow to discard documents or signatures during learning, or to determine those which are most specific for a particular disease at different points in time. One should also move from the unsupervised setting considered here to the supervised setting, for example, for classifying disease-specific spectra at different stages of pathogenesis. One approach to do so would be to train, say, a Support Vector Machine for each measurement day using our low-dimensional topic representation. A more sophisticated approach would be to adapt a temporal classifier, say, a Conditional Random Field, or to even smooth the embeddings over time using Dirichlet Multinomial Regression^37^. Ultimately, one should start developing joint models that compute low-dimensional embeddings via topics and classifications over time. This is a form of (semi)-supervised LDA, and the present work paves the way to do so.

Overall, the proposed approach will support upcoming sensor applications for phenotyping tasks like, for instance, the screening of disease resistant genotypes or precision agriculture applications for the localization of primary disease foci in fields^1^,^3^,^33^.

## Methods

### Plant material and plant pathogens

Analysis was done on a dataset recorded from barley plants which were grown in a controlled greenhouse environment and were used for hyperspectral measurements after reaching growth stage 32. A detailed description of the plant material and pathogens can be found in Wahabzada *et al*.^4^. The plants were inoculated with different fungal pathogens, namely, *Pyrenophora teres* (causing *net blotch*), *Puccinia hordei* (causing leaf *rust* of barley), and *Blumeria graminis hordei* (causing *powdery mildew*). A control group was kept non-inoculated. Hyperspectral images were recorded 4, 6, 8, 10, 12, 14 days after inoculation (dai) with an ImSpector V10E, which covers the visible and near-infrared (400–1000 nm) range. The camera has a spectral resolution of 2.8 nm and a spatial resolution of 0.12 mm per pixel, and results in 210 hyperspectral bands. The Savitzky-Golay filter^38^ was applied to remove noise and to smooth the hyperspectral signature information.

### Wordification

**Figure 1:**
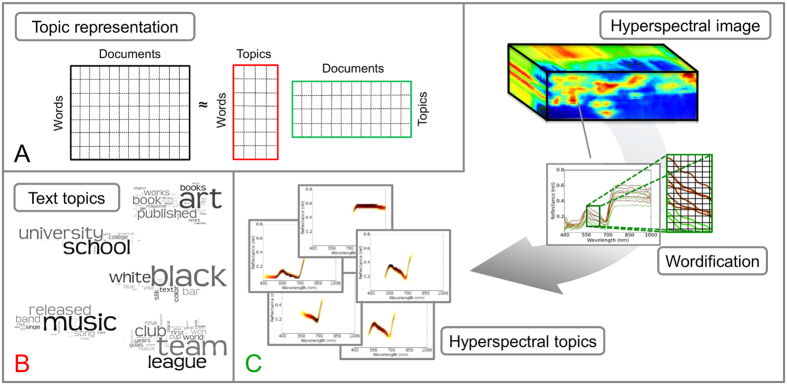
Example of interpretable matrix factorization using probabilistic topic models (**A**). It allows to represent the data (e.g. documents) as mixtures of only a few topics, which, in turn, can be learned from the data. Illustration of topics learned from text (**B**) and hyperspectral signatures (**C**) using probabilistic topic models. The text topics are represented in terms of word clouds containing words with high probabilities. The hyperspectral topics were determined using a wordification approach (**C**), and represent the spectral characteristics of healthy, diseased, and necrotic parts of leaves.

**Figure 2:**
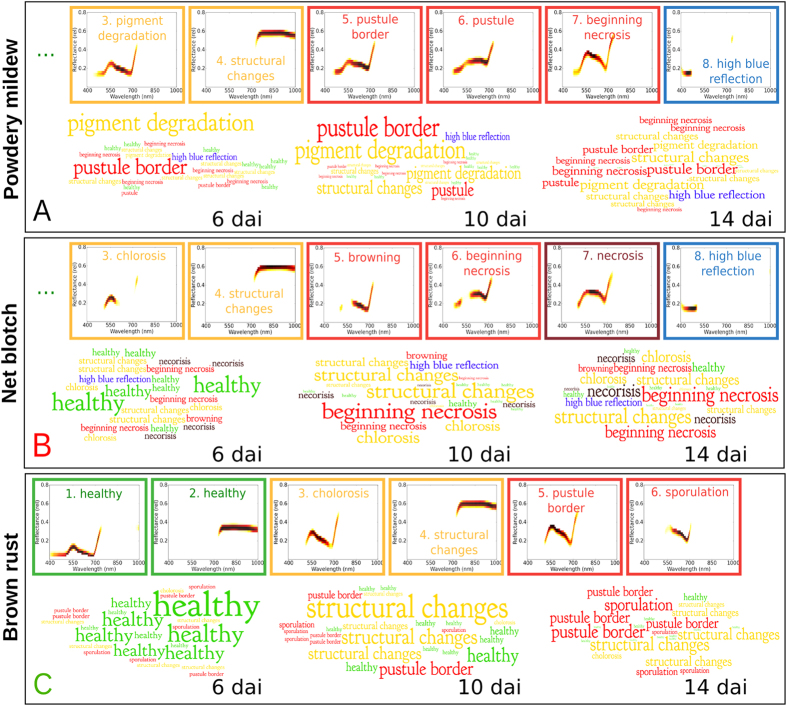
Examples of characteristic topics for different classes of plants diseased with powdery mildew, net blotch, and brown rust and topic relevance over time (6, 10 and 14 dai). Each color indicates a different class and a characteristic physiologic process, as summarized in Table 1. This approach visualizes the disease progression and relevant information from hyperspectral images. The size of the text in every second row is proportional to the computed topic relevance. The diseased/necrotic topics become more prominent at later stages, whereas the significance of healthy (green) topics is low.

**Figure 3:**
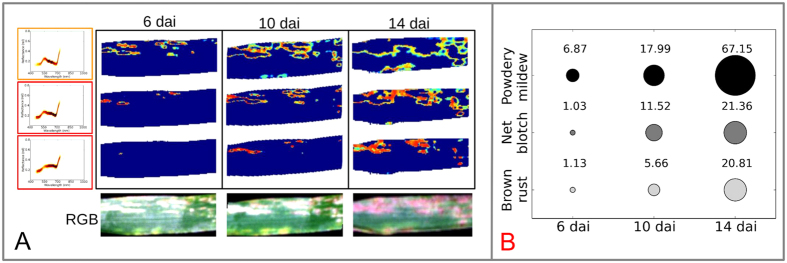
Localisation and dynamic of relevant topics of a barley leave diseased with powdery mildew at 6, 10 and 14 dai (**A**). Spatial and temporal dynamics of the topics are in accordance to the symptom development. Disease quantification based on disease specific topics 6, 10 and 14 days after inoculation (**B**). It exhibits a high sensitivity for disease detection and quantification.

We are interested in finding characteristic patterns in the combination of reflectance values at specific wavelength. To this end, we employ probabilistic topic models which require input in terms of sparse data matrices. We therefore apply wordification to given hyperspectral images. In particular, we propose to discretize hyperspectral signatures as follows: we decompose the space covering the full signatures into *R* possible reflectance words. Since reflectance values are normalized they range from 0 and 1 and thus facilitate this decomposition. Accordingly, in a signature each wavelength can consist of one of the *R* distinct reflectance words, which results in a total number of Λ × *R* different possible *spectral words*. This process is illustrated in Fig. 1(B) and detailed in Supplementary Fig. S2(A). It shows an example of a hyperspectral image where each pixel is represented by a signature. After wordification, each document (signature) is represented by Λ out of Λ × *R* possible spectral words. The benefits of this approach are that it is fast to compute, does not require additional efforts to construct a dictionary, and yields interpretable results since each word correspond to a specific wavelength-reflectance pair. The use of discretized values instead of continuous ones is further motivated by the fact that, according to plant physiologist, small difference in reflectance values are of minor importance.

Nevertheless, since signatures are still curves over spectral bands, we also take the short-range dependencies of words into account. For that we compute a word-dependency matrix *C*, cf. Supplementary Fig. S2(B), that is created using pointwise mutual information (PMI). PMI as the measure of word association is defined as follows^39^:


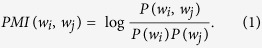


In order to also capture co-occurrences between different reflectances within a wavelength, we proceed as follows: we first aggregate the signatures in the images into non-overlapping squares of 5 × 5 pixels. *Spectral word* co-occurrences are computed using a sliding window of stride length 1 in each direction (wavelength and reflectance) in the aggregated signatures. Next, given the document-word representation of signatures and their short-range dependencies, we are ready to apply regularized topic models.

For our experiments on hyperspectral images of diseased plants, we transformed each signature into document representation by setting *R *= 50, before running the topic models. We computed the PMI only for the words with minimum appearance of *N*_*w*_ = 250 and only kept the positive values.

### Regularized Probabilistic Topic Models

LDA, as proposed by Blei *et al*.^15^, is a Bayesian probabilistic model that describes a generative process of how words in documents might be generated on the basis of latent topics. Fitting an LDA topic model given a set of training documents requires approximate inference techniques that are computationally expensive. For instance, in variational Bayesian (VB) inference, the true posterior is approximated using a simpler, fully factorized distribution *q*. For that, following^15^,^40^, we choose *q*(*z, θ, β*) of the form 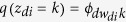, 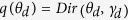, and 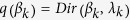. The variational parameters *ϕ, γ*, and *λ* are optimized to maximize the Evidence Lower BOund (ELBO)


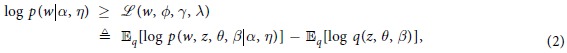


which is equivalent to minimizing the Kullback-Leibler divergence between *q*(*z, θ, β*) and the true posterior 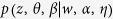.

For introducing a structured prior to regularize the word-topic probabilities, one can build on top of the recent regularized Gibbs approach due to^39^, who have demonstrated that regularization improves the topic coherence. Before presenting a scalable online approach for regularized LDA, we present a variational Bayes inference for the batch case.

### Variational Bayes Inference for Regularized LDA

For regularized LDA, we view each topic as a mixture of word probabilities given by the word-pair dependency matrix *C* (a *W *× *W* matrix, where *W* denotes the size of vocabulary and *C*_*ij*_ ≥ 0), that is


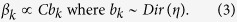


In VB, the true posterior is approximated using fully factorized distributions *q*. Consequently, we parameterize the word probabilities *b* by introducing a new variational parameter *v*, i.e. 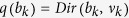. The per-word topic assignments *z* are parameterized by *ϕ*, and the posterior over the per-document topic weights *θ* are parameterized by *γ*, as for the standard LDA. The part of the likelihood including the specific parameter *v* can then be written as


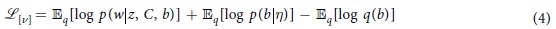


and the remaining part of the ELBO remains unchanged. To approximate the first term of Eq. (4), we adapt the lower bound on the log-sum-exp function^41^, 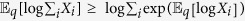 (for a detailed proof see e.g.^42^) to our case, which follows by applying Jensen’s inequality:


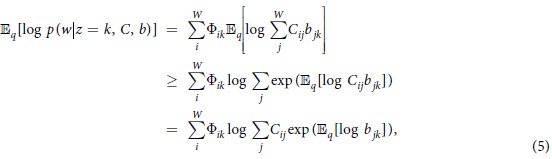


where 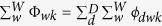. This is still a lower bound, so maximizing it will improve the ELBO. The expectation of log*b* under the distribution *q* is: 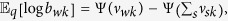 where Ψ denotes digamma function, the first derivative of logΓ (the logarithm of the gamma function). The remaining terms of the Eq. (4) (for a topic k) are


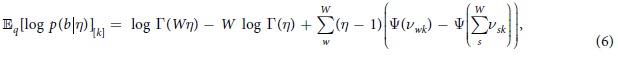



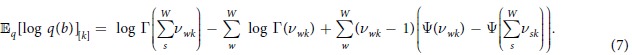


To derive a VB approach, we compute the derivative of Eq. (4) with respect to the variational parameter *v*_*wk*_. After applying the chain rule and rearranging terms, this gives


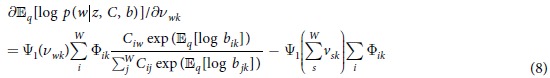


for the first term of Eq. (4). Taking the derivatives for all terms together we arrive at:


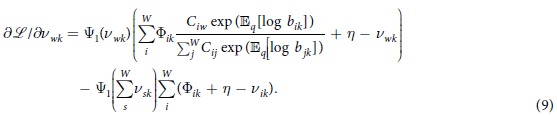


Setting the above derivative to zero, we obtain the following fixed point update:


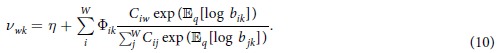


This is a proper generalization of the standard VB approach. To see this, we simply set the word-pair dependency matrix *C* to the identity matrix. It then follows that


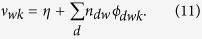


To derive a learning algorithm, i.e. to actually optimize 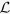, we follow a coordinate ascent on the variational parameters *ϕ, γ* and *v*. Given the word topic probabilities *β* from Eq. (3), this yields the following per-document updates for *ϕ* and *γ* in the E-step:


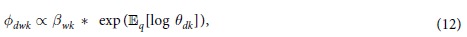



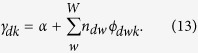


In the M-step, we perform fixed point updates as in Eq. (10) and compute the values *β*_*wk*_ using Eq. (3) as follows:


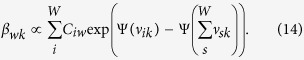


However, recall that one of our main goals is the application of regularized VB to hyperspectral images of plants. Since a single image can already consist of hundreds of thousands of signatures (documents) so that several images (as in the case of our experiments) easily scale to several million documents, batch VB is likely to be infeasible in terms of running time. Consequently, we will develop an online variant of regularized VB that scales well to massive datasets.

### Online Variational Bayes Inference for Regularized LDA

**Figure 4:**
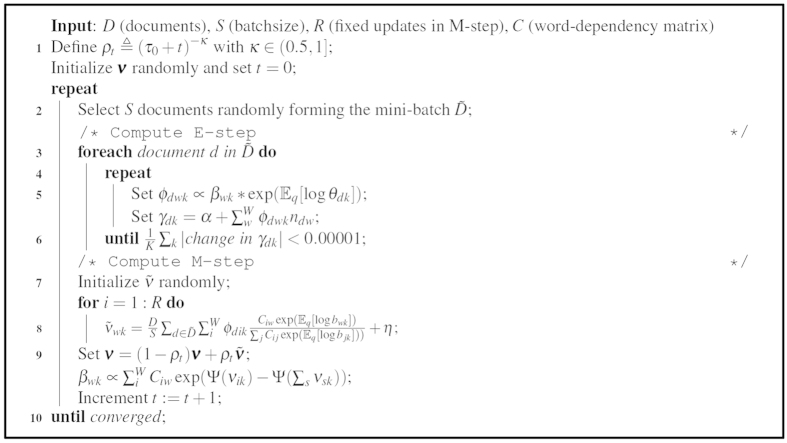
Online variational Bayes for regularized latent Dirichlet allocation.

Since setting the word-dependency matrix *C* to identity matrix results in standard VB, it is intuitively clear that we may extend the regularized VB to the online case by adapting online variational Bayes^40^. Specifically, the variational lower bound for the regularized VB can be written as


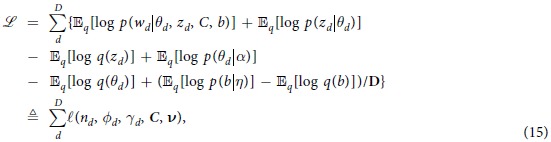


where 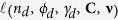 is the *dth* document’s contribution to the variational bound. The per-corpus terms are summed together and divided by the number of documents *D*. This allows us to derive the online approach since the optimal *v* is the one for which 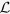 maximized after fitting the per-document parameter. In other words, we can use the regularized updates in a per-document manner as summarized in Fig. 4.

The algorithm first randomly selects documents from the entire dataset by forming a mini-batch 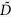. Then, an E-step is performed to find locally optimal values of *γ* and *ϕ* while holding *β* fix. In the M-step, several fixed point updates for 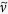 are computed using


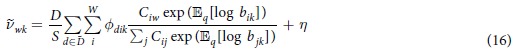


given the document-specific parameter *ϕ*_*d*_ with 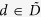 (currently observed mini-batch), where we re-scale by 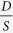 to update as though we would have seen all documents. Multiple documents are used per update to reduce variance. The parameter 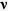 is updated through a weighted average of its previous value, and 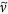 (computed for the current mini-batch using fixed point updates as in Eq. (16)). Furthermore, new values of *β* are computed given 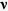 and word-dependency matrix *C*. The rate of change 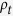 is set to 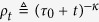 with 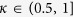 in order to guarantee convergence. Note that, as in the non-regularized case, when setting the batch size to *S* = *D* and 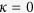 we recover regularized batch VB.

## Additional Information

**How to cite this article**: Wahabzada, M. *et al*. Plant Phenotyping using Probabilistic Topic Models: Uncovering the Hyperspectral Language of Plants. *Sci. Rep.* **6**, 22482; doi: 10.1038/srep22482 (2016).

## Supplementary Material

Supplementary Information

## Figures and Tables

**Table 1:** Relevant spectral topics and corresponding biochemical labels in the visble and near-infrared range.

Disease	Class	Label	Relevant functional spectral range	Literature	Description and symptom apparance
Powdery mildew *Blumeria graminis hordei*	1	healthy VIS	400–700 nm, partly 700–1000 nm	27,43,44	green, healthy leaf tissue with high pigment absorbance
	2	healthy NIR	700–1000 nm	45,46	healthy tissue with moderate backscattering
	3	pigment degradation VIS	500–650 nm	27,44,47	beginning chlorosis, outer border of pustules
	4	structural changes NIR	700–1000 nm	45,48,49	mycelium growth and development of conidiophore and conidia causing increased backscattering
	5	pustule border	560–700 nm	47	browning, inner border pustules
	6	pustule	560–700 nm	10,27,44	high VIS reflectance / shift green peak
	7	beginning necrosis	500–680 nm	47	beginning necrosis at pustule sites, center pustules
	8	high blue reflection	400–450 nm	50	powdery mildew mycelium, conidiophores and conidia
Net blotch *Pyrenophora teres*	1	healthy VIS	400–700 nm, partly 700–1000 nm	27,43,44	green, healthy leaf tissue with high pigment absorbance
	2	healthy NIR	700–1000 nm	45,46	healthy tissue with moderate NIR reflectance
	3	chlorosis VIS	500–580 nm, 550 nm, 700 nm	27,44,47	pigment degradation and chlorosis at symptom sites
	4	structural changes NIR	700–1000 nm	45,48	beginning tissue damage
	5	browning	580–700 nm	47	net-like symptom development
	6	beginning necrosis	580–700 nm	51	inner parts of the symptoms with characteristic net-like necrosis
	7	necrosis	450-700 nm, 680 nm	47	tissue damage and drying causing shift of the red edge
	8	high blue reflection	400–500 nm	50	increased blue reflection
Brown rust *Puccinia hordei*	2	healthy VIS	400–700 nm partly 700–1000 nm	27,34,52	green, healthy leaf tissue with high pigment absorbance
	1	healthy NIR	700–1000 nm	45,50	healthy tissue with moderate NIR reflectance
	4	chlorosis VIS	550–650 nm	43,44,53	chlorotic halos around rust pustules
	3	structural changes NIR	700–1000 nm	45,48	increased NIR plateu caused by ruptured epidermis and tissue damage
	5	pustule border	550–650 nm	43	first uredospores appear, inner border pustules
	6	sporulation	600–710 nm	43	uredospores appear at the center of rust pustules, advanced senescence
