# Supplementary material for: Plant Phenotyping using Probabilistic Topic Models: Uncovering the Hyperspectral Language of Plants
Source: Sci Rep. 2016 Mar 9;6:22482. doi: 10.1038/srep22482 (PMC4783663; doi:10.1038/srep22482)
Supplement: Supplementary Information [file srep22482-s1.pdf]

Plant Phenotyping using Probabilistic Topic Models: Uncovering the Hyperspectral Language of Plants

Mirwaes Wahabzada[1,*,+], **Anne-Katrin Mahlein**[1,+], **Christian Bauckhage**[2,3], **Ulrike Steiner**[1], **Erich-Christian Oerke**[1], **and Kristian Kersting**[4]

[1]INRES-Phytomedicine, University of Bonn, Bonn, Germany
[2]Fraunhofer IAIS, Sankt Augustin, Germany
[3]B-IT, University of Bonn, Bonn, Germany
[4]CS Department, Technical University of Dortmund, Dortmund, Germany
[*]mirwaes@uni-bonn.de
[+]these authors contributed equally to this work

Supplementary Information

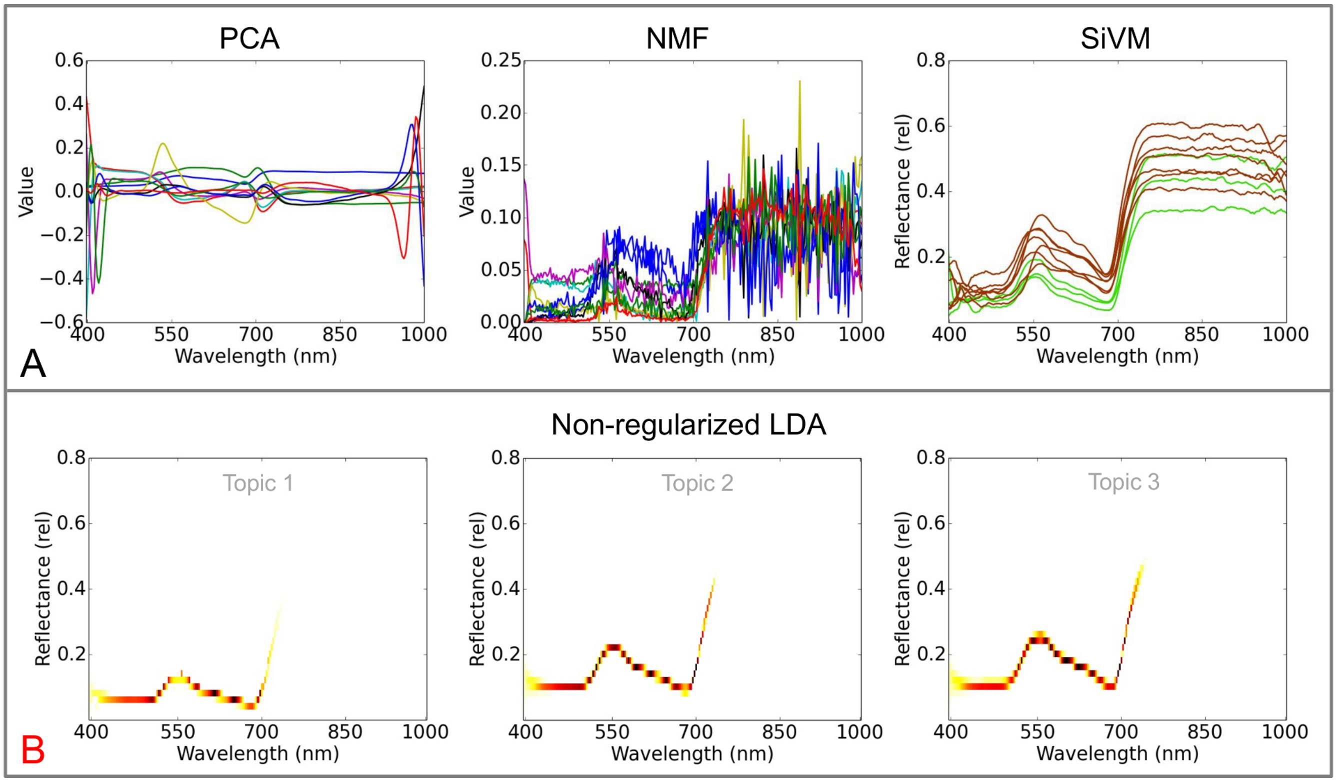

Figure S1. Latent components determined from a hyperspectral image of a diseased plant with brown rust by different methods. Components obtained by PCA, NMF and SiVM are determined using original representation of reflectance information (A). These methods find components that are not easy to interpret (PCA), are corrupted by noise and difficult to understand (NMF) or do not represent important parts of the spectrum (SiVM). In contrast to regularized LDA, the topics found by non-regularized LDA on sparse data obtained by wordification (B) are not coherent and are dominated by the topics with lower reflectances, which represent the healthy part of the leaf, by ignoring the variations of diseased spectra.

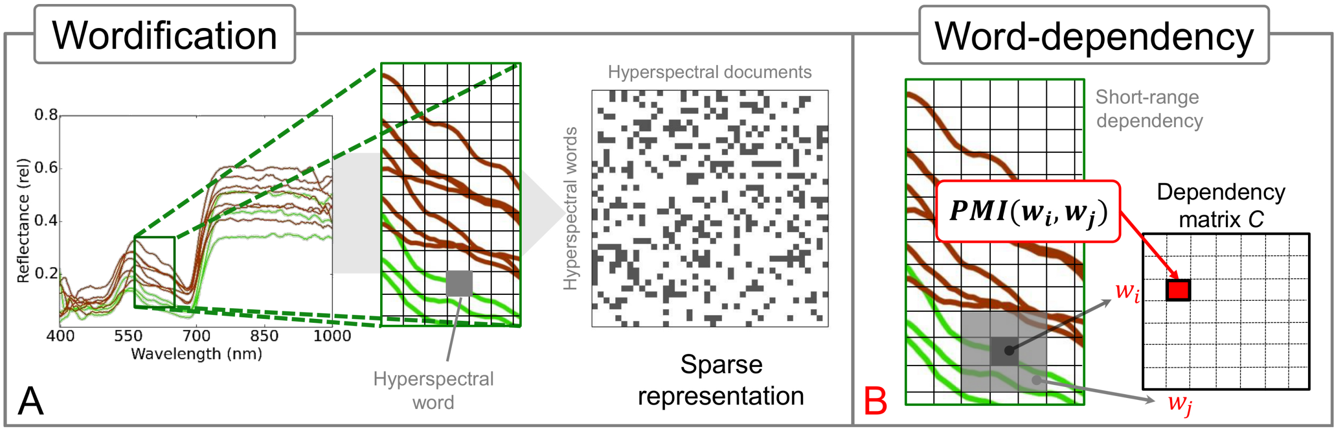

Figure S2. A wordification approach for hyperspectral images (A): Discretize the space covered by signatures into R reflectance words, whereas each square denote a hyperspectral word. The corresponding sparse representation, after the wordification was applied, represents each hyperspectral signature (curve) as sparse vector which can be used to learn a topic model. (B) Construction of word-dependency matrix C using word co-occurrences and pointwise mutual information. The matrix is used within online regularized topic model to discover coherent topics.
